# Plasma Orexin-A Levels in COPD Patients with Hypercapnic Respiratory Failure

**DOI:** 10.1155/2011/754847

**Published:** 2011-05-26

**Authors:** Lin-Yun Zhu, Hanssa Summah, Hong-Ni Jiang, Jie-Ming Qu

**Affiliations:** ^1^Department of Pulmonary Medicine, Zhongshan Hospital, Fudan University School of Medicine, Shanghai 200032, China; ^2^Department of Pulmonary Medicine, Huadong Hospital, Fudan University School of Medicine, Shanghai 200040, China

## Abstract

Orexins have previously been shown to promote wakefulness, regulate lipid metabolism and participate in energy homeostasis. The aim of the study was to determine the relationship between plasma orexin-A and body composition in COPD in-patients with hypercapnic respiratory failure. 40 patients with hypercapnic respiratory failure and 22 healthy individuals were enrolled prospectively in this study. Plasma orexin-A levels, BMI, SaO_2_, PaCO_2_ and PaO_2_ were noted for all the patients. Plasma orexin-A levels were higher in the underweight (UW) group, normal weight (NW) group and overweight (OW) group of COPD patients as compared with UW, NW and OW group of the control group (*P* < .05). Plasma orexin-A in COPD patients were higher in the OW group than in the NW group and the UW group. Plasma orexin-A levels showed significant correlation with body mass index (BMI), independent of PaO_2_ (*r* = 0.576; *P* < .05) and %fat (*r* = 0.367; *P* < .05); a negative correlation was noted between plasma orexin-A levels and PaO_2_ (*r* = −0.738; *P* < .05) and SaO_2_ (*r* = −0.616; *P* < .05). Our results suggest that orexin-A levels are high in COPD patients with hypercapnic respiratory failure, and vary according to BMI and body composition. Orexin-A may be associated with the severity of hypoxemia in COPD patients with hypercapnic respiratory failure.

## 1. Introduction

Body weight is an important prognostic factor of chronic obstructive pulmonary disease in that a low body weight correlates with increased morbidity and poor prognosis [1–3]. This is mainly because a low body weight in COPD patients implies weakening of both respiratory muscles and skeletal muscles [4, 5]. The weakening of respiratory muscles, in turn, makes COPD patients vulnerable to respiratory failure. It is widely known that COPD is one of the main causes of acute respiratory failure. Resting hypoxia, usually due to *V*_*A*_/*Q* mismatching, and exercise aggravation of hypoxia due to increased flow and oxygen disequilibrium are often seen in COPD patients [6, 7]. Acute hypercapnic respiratory failure as a result of an acute exacerbation of COPD is a common reason for emergency hospital admission [8]. It has been noted that an acute exacerbation of COPD entails decreased food intake and increased resting energy expenditure, thus causing energy disbalance in these patients [9]. 

 Orexin-A and orexin-B belong to the family of orexins are neuropeptides identified within the lateral hypothalamus during the last decade [10]. Previous studies show that intraventricular injection of orexin-A not only produces a dose-dependent food intake but also increases the metabolic rate [10, 11]. Apart from promoting wakefulness and regulating lipid metabolism, orexin-A has been implicated in diabetes mellitus and obesity [12–14]. In 2003, Matsumura et al. successfully showed that plasma orexin-A levels are lower in COPD patients, with a lower level being more prominent in underweight patients as compared to normal individuals [15]. However, whether plasma orexin-A levels show the same variation in COPD patients with hypercapnic respiratory failure has not been investigated. We therefore tried to assess plasma orexin-A levels during an acute exacerbation of COPD.

## 2. Materials and Methods

### 2.1. Subjects

Forty COPD patients with hypercapnic respiratory failure who were hospitalized in the Department of Pulmonary Medicine of Zhongshan Hospital, Shanghai, were included in the study. The diagnosis of COPD was based on past smoking history, clinical evaluation, and pulmonary function tests according to the American Thoracic Society Guidelines [16]. All the 40 patients had an arterial partial pressure of CO_2_ (PaCO_2_) of ≥50 mmHg, which is line with hypercapnic respiratory failure diagnosis. Patients with a history of use of systemic corticosteroids or with a history of sleep-related breathing disorders, malignancy, cardiac failure, diabetes mellitus, or other endocrine diseases were excluded. The control group consisted of 22 healthy individuals, without any underlying disease and with normal pulmonary function as defined by (FEV_1_/FVC > 70%) who presented to the Zhongshan Hospital for routine physical examination. The research protocol was approved by the Ethics Committee of Fudan University, and written consent form was obtained for each subject.

### 2.2. Nutritional Assessment

The following parameters were recorded for each patient: age (in years), body weight (in kilograms), and height (in meters). Based on these measures, body mass index (BMI) and body fat percentage were calculated. BMI was calculated as weight/(height)^2^. Body fat % was calculated as follows: male = (1.2 × BMI) + (0.23 × age) − 16.2, and female = (1.2 × BMI) + (0.23 × age) − 5.4. 

 Based in their BMI, the control group subjects and the patients with COPD were further subdivided into 3 groups: (1) underweight (UW) group (BMI < 20), (2) normal weight (NW) group (20 ≤ BMI < 25), and (3) overweight (OW) group (BMI ≥ 25).

### 2.3. Samples

4 mL blood samples were collected from the antecubital vein by using a vacuum blood-collecting tube containing aprotinin at noon. Plasma, separated by centrifugation, was stored at −80°C until analysis. Orexin-A concentrations were always measured in duplicate in the following way: plasma orexin-A was first extracted using extraction columns (Extra-Sep C18, Lida, Kenosha, Wisc., USA). Then, column eluants were evaporated to dryness in a centrifugal concentrator and reconstituted in radioimmunoassay buffer. Finally, orexin-A levels in the reconstituted aliquots were determined using an iodine-125 hypocretin-1 RIA system (Phoenix Pharmaceuticals, Mountain View, Calif, USA). Mean values of orexin-A were calculated and used for statistical analysis. 

 Arterial blood was drawn from all subjects during room air breathing at the same time that blood was collected for orexin-A level measurement.

### 2.4. Statistical Analysis

The Mann-Whitney *U*-test and ANOVA were used to analyze the difference between two groups. Spearman's rank correlation technique and partial correlation technique were used to analyze the relationship between several continuous variables. Results are expressed as medians [range] or mean ± SE as appropriate. Statistical analysis was performed using a statistical software package (SPSS 13.0); *P* < .05 was considered to be statistically significant.

## 3. Results

### 3.1. Subject Characteristics

Out of the 40 patients with COPD, 32 were male and 8 were female. The mean age of the subjects was (74.2 ± 8.7) years and the mean BMI was (21.7 ± 3.0) kg/m^2^. Out of the 22 control subjects, 17 were male and 5 were female. The mean age was (73.1 ± 10.1) years and the mean BMI was (22.3 ± 3.0) kg/m^2^. The mean age and the mean BMI of COPD patients and control subjects belonging to the UW, NW, and OW groups are shown in Table 1. There was no statistical difference in the baseline characteristics (age and BMI) between the patients with COPD and the control subjects.

### 3.2. Plasma Orexin-A Levels

Plasma orexin-A levels in each group of COPD patients and control subjects are shown in Table 2. UW, NW, and OW patients with COPD had significantly higher plasma orexin-A levels as compared to UW, NW, and OW control subjects (*P* < .000, *P* < .000, and *P* < .001, resp.). 

### 3.3. Relationship between Different Observed Indexes of COPD Patients and Control Subjects and Plasma Orexin-A Levels

**Figure 1:**
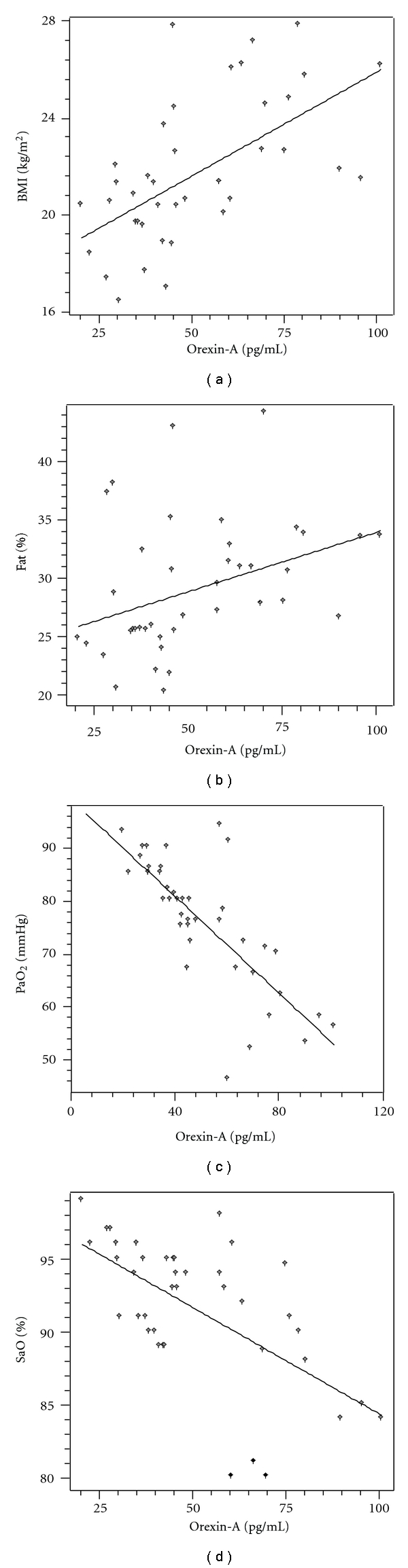
(a) Relationship between BMI and orexin-A. (b) Relationship between %fat and orexin-A. (c) Relationship between PaO_2_ and orexin-A. (d) Relationship between SaO_2_ and orexin-A. In patients with COPD, plasma orexin-A levels significantly correlated with BMI (*r* = 0.589, *P* = .000), %fat (*r* = 0.367, *P* = .020), PaO_2_ (*r* = −0.738, *P* = .000), SaO_2_ (*r* = −0.616, *P* = .000).

Plasma orexin-A levels in COPD patients were found to correlate significantly with BMI (*r* = 0.589, *P* = .000) (Figure 1(a)), %fat (*r* = 0.367, *P* = .020) (Figure 1(b)), PaO_2_ (*r* = −0.738, *P* = .000) (Figure 1(c)), and SaO_2_ (*r* = −0.616, *P* = .000) (Figure 1(d)). Partial correlation analysis was used to determine whether the correlation between plasma orexin-A and BMI was independent of the degree of respiratory failure, and, controlling PaO_2_, plasma orexin-A was still found to correlate significantly with BMI (*r* = 0.576, *P* = .000). However, no correlation was found between PaCO_2_ and plasma orexin-A levels (*P* = .173). 

 Plasma orexin-A levels in control subjects were found to correlate significantly with BMI (*r* = 0.428, *P* = .047).

## 4. Discussion

Orexin, also known as hypocretin, was first described in 1998 by de Lecea et al. [17]. In 1999, orexin neurons were found to be present in the lateral hypothalamic area, perifornical nucleus, diffuse part of the dorsomedial hypothalamic nucleus, and posterior hypothalamus [18]. It has been previously shown that orexins can pass the blood-brain barrier by simple diffusion [19] and that orexins and orexins receptors are present in the hypothalamus as well as the enteric nervous system, adipose tissue, the pancreas and the gut [20, 21]. Orexin not only acts as an appetite stimulator but also acts along with other neuropeptides to regulate feeding behavior [22, 23]. 

Our results show that plasma orexin-A levels in COPD patients with hypercapnic respiratory failure are much higher than those in normal subjects. These results differ from those of Matsumura et al. [15]. We can explain the discrepancies in our results by the fact that our study included a larger number of patients and our patients were all hospitalized COPD patients with hypercapnic respiratory failure while those of Matsumura et al. were outpatients, without any exacerbation of COPD. COPD patients with hypercapnic respiratory failure have a larger amount of energy expenditure and have decreased food intake as compared to COPD patients who are not suffering from an acute disease exacerbation. This energy imbalance may well be another plausible explanation for the higher values of plasma orexin-A in COPD patients in our study. Due to the fact that COPD patients with hypercapnic respiratory failure restricted food intake, there is subsequently an increase in the expression of prepro-orexin gene [24], which in turn causes an increase in the plasma orexin-A levels noted in our patients.

Patients from the OW group were found to have higher plasma orexin-A levels than those from the NW group, who in turn, had higher plasma orexin-A levels than those of the UW group. We further found that plasma orexin-A levels and BMI were correlated and the correlation was independent of the degree of respiratory failure. These results are in concordance with those of Matsumura et al. Other previous studies have shown that plasma orexin-A levels correlate with BMI [25, 26]. However, Adam et al. found that plasma orexin-A was lower in obese individuals [27]. These different results might well be explained by the fact that plasma orexin-A levels might be dependent on the underlying disease from which the patients are suffering from. In our present study, we also found a positive correlation between plasma orexin-A levels and %fat in COPD patients with hypercapnic respiratory failure. In 2002, Wortley et al. showed that circulating lipids increase in obesity and the increase in triglyceride levels increase hypothalamic orexin gene expression in rats [28]. In 2006, Digby et al. showed that orexin receptors are found in adipose tissue and that orexins may play a role in adipogenesis [21]. Therefore, our results are in concordance with these findings.

Orexin participates in respiratory control by increasing ventilation [29]. Dreshaj et al. showed that lesions in the basomedial hypothalamus have been found to influence respiratory response in neonatal rats in that the latter show impaired response to hypoxia. However, these lesions did not affect the hypercapnic responses [30]. On the other hand, Deng et al. showed that orexin knock-out mice had weakened respiratory chemoreflex to hypercapnic gases, but not to hypoxic gases [31]. In our study, we found no correlation between plasma orexin-A levels and PaCO_2_; but, a significant negative correlation was noted between plasma orexin-A levels and PaO_2_ and SaO_2_. Similar findings have been formerly noted [32]. Our results indicate that orexin-A may play an important part during hypoxia; however, the underlying mechanism remains to be revealed. 

Here we must point out that there are several limitations to our study. Firstly, we included a limited number of patients. Although plasma orexin-A levels in COPD patients were found to correlate significantly with BMI, %fat, PaO_2_ and SaO_2_, the results for individual COPD patients were in fact, quite variable, and therefore, studies including larger number of patients should be carried out in order to confirm our results. Moreover, in our study we did not study the mechanism through which orexin-A is upregulated during hypoxia.

## 5. Conclusions

This study shows that plasma orexin-A levels correlate with BMI and %fat in COPD patients with hypercapnic respiratory failure and that orexin-A levels may be altered during hypoxia, and more studies are required to determine the role of orexin-A in hypoxia.

##  Conflict of Interest 

None of the authors have any conflict to disclose.

## Figures and Tables

**Table 1:** Baseline characteristics of subjects.

Group		*n*	Age (years)	BMI (kg/m^2^)
Control subjects	UW	5	73.8 ± 11.9	19.2 ± 0.9
	NW	14	72.6 ± 10.4	22.0 ± 1.1
	OW	3	74.0 ± 8.7	28.5 ± 2.4

COPD	UW	10	76.2 ± 5.0	18.3 ± 1.2
	NW	23	73.2 ± 10.4	21.7 ± 1.4
	OW	7	74.7 ± 7.0	26.2 ± 0.9

**Table 2:** Plasma orexin-A levels in weight-based group.

	Control subjects			Patients with COPD			
	*n*	orexin-A (pg/mL)		*n*	orexin-A (pg/mL)		*P*
		median	range		median	range	
UW	5	9.00	8.00–18.69	10	36.32	22.71–44.88	.000
NW	14	15.44	5.96–33.61	23	48.51	14.55–95.86	.000
OW	3	20.55	10.18–23.62	7	56.00	45.26–101.26	.001
